# Emergency peripartum hysterectomy in the Dubai health system: A fifteen year experience

**DOI:** 10.4274/tjod.55492

**Published:** 2018-03-28

**Authors:** Muna Abdulrazzaq Tahlak, Mahera Abdulrahman, Nawal Mahmood Hubaishi, Mushtaq Omar, Fatima Cherifi, Shazia Magray, Frederick Robert Carrick

**Affiliations:** 1 Dubai Health Authority, Latifa Women and Children Hospital, Clinic of Obstetrics and Gynegology Dubai, United Arab Emirates; 2 Dubai Health Authority, Clinic of Medical Education, Dubai, United Arab Emirates; 3 Dubai Health Authority, Dubai Hospital, Clinic of Obstetrics and Gynegology, Dubai, United Arab Emirates; 4 Bedfordshire Centre for Mental Health Research in Association with University of Cambridge, Department of Neurology and Senior Research, Cambridge, United Kingdom; 5 Harvard Medical School-Harvard Macy Institute, Clinic of Nevrology, Boston, USA; 6 Carrick Institute, Cape Canaveral, Clinic of Nevrology, Florida, USA

**Keywords:** Emergency hysterectomy, peripartum hysterectomy, abnormal placentation, risk factors

## Abstract

**Objective:**

To determine the incidence, demographic data, risk factors, indications, outcome and complications of emergency peripartum hysterectomy (EPH) performed in two major tertiary care hospitals in Dubai, and to compare the results with the literature.

**Materials and Methods:**

The records of all women who underwent EPH from January 2000 to December 2015 in two major tertiary care hospitals in Dubai were accessed and reviewed. Maternal characteristics, hysterectomy indications, outcomes, and postoperative complications were recorded using descriptive statistics to describe the cohort.

**Results:**

There were 79 EPH out of 168.293 deliveries, a rate of 0.47/1000 deliveries. The most common indications for hysterectomy were abnormal placentation (previa and/or accreta) and uterine atony. The majority of hysterectomies were subtotal (70%). The complications were dominated by massive transfusion, urinary tract injuries, one case of maternal death, and one case of neonatal death.

**Conclusion:**

The main indication for EPH was abnormal placentation in scarred uterus and uterine atony. The major method of prevention of EPH is to assess women’s risks and to reduce the number of cesarean section deliveries, by limiting the rate of primary cesareans. This is challenging in the United Arab Emirates (UAE) where the culture is for high gravidity and high parity. Recommendations to act to reduce primary and repeated cesareans should be included on the national agenda in UAE.

**PRECIS:** The purpose of this study was to review the incidence of cesarean sections, risk factors, indications, medical and surgical management, and the outcomes of peripartum hysterectomy over the past 15 years in Dubai.

## Introduction

Emergency peripartum hysterectomy (EPH) is an uncommon obstetric procedure performed after 20 weeks of gestation and within twenty-four hours of birth, usually performed as a life-saving measure in cases of intractable obstetric hemorrhage. We could not find any epidemiologic evidence of the incidence rate of EPH in the United Arab Emirates (UAE) and desired to know if it was similar to the estimated 1.5 per 1.000 deliveries that have been reported in countries with modern health care facilities^(1)^. Dubai won the “Highly Recommended Destination of the Year” award from the Medical Travel Journal in 2016 and is actively becoming a medical tourism destination expecting more than one million medical tourists by 2020^(2)^. Many of these tourists will be pregnant and we are concerned with the world wide increase in EPH and our need to understand its impact on all patients and the health system in the UAE. We realize that postpartum hemorrhage (PPH) remains a significant threat to maternal outcomes despite technological and pharmacologic advances. Globally, obstetric hemorrhage is still the leading cause of maternal mortality^(3)^ and therefore peripartum hysterectomy poses a challenging complication for obstetricians given the high risk of maternal death and morbidity^(4)^. We understand that management of EPH is complicated by the need for massive blood transfusions, injury to the urinary tract, coagulopathy, and the need of re-exploration, followed by prolonged hospitalization^(5,6,7)^. Throughout the world, the factors necessitating EPH include uterine atony and uterine rupture, but this has been largely overtaken by abnormal placentation, which is now the most common indication for peripartum hysterectomy^(3,8,9)^. We identified a need to know if similar factors were involved in the UAE so that we might prepare for and include improved conservative methods to treat obstetric hemorrhage that might reduce the incidence and complications of uterine rupture. Abnormal placentation involving placenta previa and the morbidly adherent placenta followed a global rise in cesarean section (CS) rates over the past two decades. We needed to address the rates of CS in medical tourists and our local population. We know that uterine scarring associated with abnormal placentation has been reported to increase the risk of EPH following previous CS^(10,11)^. Given the socio-economic differences among the Arab countries in the Middle East, the UAE has a constant patient influx from neighboring countries as well as medical tourists. In the UAE, Dubai in particular, one of the two large emirates, stands out as an affordable quality healthcare provider. The modern healthcare infrastructure, physician expertise, and multi-ethnic healthcare providers make Dubai attractive for health tourism. High-risk cases, such as multiple CS and multiparity are commonly handled by the Dubai Health Authority (DHA), a public sector healthcare provider in Dubai with four hospitals and 16 primary health care centers. Dubai public hospitals, governed by the DHA are accredited by the Joint Commission International and serve as teaching hospitals for medical students and residency training^(12,13)^. A vast majority of the physicians in the DHA network are either trained in the United States or Europe. The DHA mandates a multidisciplinary team approach for the management of women with abnormal placentation, such as the placenta accreta team, which includes radiologists, feto-maternal medicine specialists, urologists, vascular surgeons, gynecologic oncologists and an anesthetist. The neonatal intensive care unit (NICU) has maintained an affiliation with the Oxford Vermont network since 2010. In spite of the modern healthcare system in Dubai, unregistered women without antenatal care and without referrals frequent the DHA maternity hospitals. Although the exact proportion of these women is not known, we expect that they may make up 20% of the total maternity patients. These and other challenges prompted us to investigate the rate of CS, EPH, and associated factors in women attending DHA hospitals. The purpose of this study is to review the incidence of CS, risk factors, indications, medical and surgical management, and the outcomes of peripartum hysterectomy over the past 15 years in DHA maternity hospitals.

## Materials and Methods

### Study population

This study was approved by the Research Ethics Committee of the DHA (approval number: DSREC-12/2015_15). Consent was not needed for retrospective studies as per the DHA ethics committee. The Latifa Hospital (LH) and Dubai Hospital (DH) are specialized tertiary hospitals for women and children governed by the DHA. Both hospitals serve as referral centers for high-risk, complicated cases within the country and the region. On average, 10.000 deliveries per year occur in both hospitals combined. Both LH and DHs are considered multidisciplinary care centers, defined as institutions with 24-hour in-house obstetrician-gynecologists, anesthesiologists, fully-stocked blood banks, immediate availability of a gynecologic oncologist, vascular surgeon, and a urologist. The study was conducted at these two hospital sites and our design was a retrospective cohort including all pregnancies complicated by EPH over a 15-year period from January 2000 until December 2015. The International Classification of Diseases-9 code was used to extract data from all hysterectomy cases, which included placenta accreta, postpartum hysterectomy, PPH (immediate and delayed), postpartum coagulation defects, and retained placenta or membranes (with or without complications or hemorrhage or both) from the hospital information system by the health informatics department. Peripartum hysterectomy, defined as a woman who had a hysterectomy for a hemorrhage that was unresponsive to all other treatment modalities served as the inclusion criteria. Deliveries of less than 24 weeks gestation were excluded from the study. Data extraction was independently performed by two extractors and variations in data were subjected to the interrater reliability test.

### Demographic data, risk factors, indications, and outcome variables

After the records had been obtained and de-identified, a matrix database was assembled. We identified maternal sociodemographic details, past medical, surgical and obstetrical histories, labor and delivery events, including gestational age, mode of delivery, indications of CS and type of hysterectomy performed. We also reviewed any additional procedures performed, blood loss, blood transfusions, and postoperative complications. PPH was defined as blood loss of 500 mL or more from the genital tract within 24 hours of the birth of a baby. Antepartum hemorrhage was defined as bleeding from or into the genital tract occurring from 24 weeks of pregnancy and before the birth of the baby. Blood loss was estimated by evaluating the woman’s hemodynamic status, serial intraoperative hemoglobin by the anesthetist, blood-soaked swabs and linen, and blood collected in the kidney trays. The numbers of units of blood transfused, the pathology reports, and maternal complications including maternal death and urologic, infectious and wound complications were evaluated after the operation. Blood transfusions were calculated by the number of units of fresh frozen plasma and whole blood given during hospitalization. Placenta accreta is defined as the placenta being adherent to the uterine wall without easy separation and included the spectrum of placenta accreta, increta, and percreta^(11)^. The diagnoses were suspected by ultrasound/magnetic resonance imaging findings and confirmed by histopathologic-evidence of placental invasion into the myometrium, by clinical assessment of abnormal adherence of the placenta, or by evidence of gross placental invasion at the time of surgery. The cases were subjected to chart abstraction for maternal medical, obstetric, and gynecologic history; the timing of diagnosis; antepartum and intrapartum management; maternal postpartum course; and complications occurring within the postpartum period. Delivery was considered “elective” if planned at least 24-hours in advance and performed not urgently because of either documented fetal lung maturity or clinical concerns for risks associated with expectant management such as eventual hemorrhage or labor. The patient (woman) was considered “registered” if she had previous antenatal visits, and “unregistered” if she had no prenatal visits before and seen first time during the admission.

### Statistical Analysis

Descriptive statistics were used to describe the cohort. Student’s t-test, χ^2^ analysis, and Fisher’s exact test were used as appropriate. The Mann-Whitney U test was used to compare medians between groups for nonparametric data. SPSS Statistical Software Version 23 (SPSS Inc. Chicago, USA) was used for this purpose.

## Results

**Figure 1:**
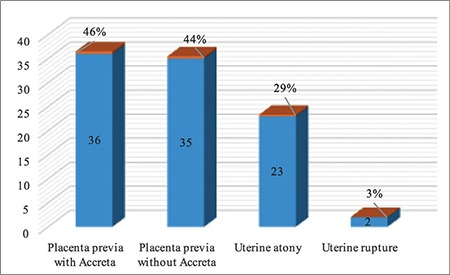
Indications of emergency peripartum hysterectomy in Dubai health authority

**Figure 2:**
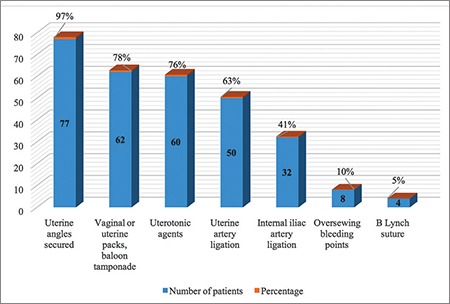
Interventions made prior to emergency peripartum hysterectomy

A total of 168.293 deliveries were performed at LH and DH with 44.376 (26%) CSs and 79 cases of EPH, during the 15-year period. The incidence of EPH was 0.47 per 1000 deliveries (Table 1). The mean age of the patients with EPH was 33.5±4 years (range, 22-41 years), 43 (54%) were UAE nationals, 66 (83.5%) were housewives, all were married, with a mean body mass index of 29.7±6 kg/m^2^ (range, 18.9-56.8 kg/m^2^). The mean gestational age was 33.7±3.8 weeks (range, 21-41 weeks) with a mean birth weight of 2366±828 g (range, 610-4500 g), and the mean parity was 3.2±1.9 (range, 0-10) for the EPH group. The general characteristics of the women with EPH are presented in Table 2. Cohen’s kappa yielded a score of 0.82, indicating acceptable inter-rater reliability (data not shown). All hysterectomies were performed within 24 hours after delivery, and all the women received prophylactic antibiotics. The majority of the hysterectomies (n=55, 70%) where subtotal. The mean number of postoperative hospitalization days was 16±14 days (range, 4-66 days), the median operating time was 125 minutes (range, 62-230 minutes), the mean estimated blood loss during PPH was 5.2±3.4 L with a range between 0.5 to 20 L. Out of 79 women, 77 (98%) births were delivered through CS, and two had vaginal deliveries, one of which was complicated by uterine atony and another by uterine rupture. Previous CS (one or more) had been performed in 69 women (87%), 71 (93%) had placenta previa associated or not with placenta accreta. Nineteen (20%) women with EPH had a previous curettage,  16 of which were associated with one or more CS. Abnormal placentation was reported in 71 (93%) patients (Table 2). There was a significant association between many previous CSs and abnormal placentation (p=0.008). Although abnormal placentation has been seen more frequently in multiparous women (72/74), this association was not statistically significant (p=0.614). No association was detected between abnormal placentation and previous uterine procedures (p=0.183). The operative notes and the pathology reports of the uterus and placenta were used to determine the final indication for the procedure. The most common indications were placenta previa with accreta 36 (46%), placenta previa without accreta 35 (44%), uterine atony 23 (29%), and uterine rupture 2 (3%) (Figure 1). Twenty-nine (37%) women had antepartum hemorrhage, whereas 71 (90%) were diagnosed as having placenta previa with or without accreta. There were 76 (96%) multiparous and 3 (4%) primiparous women. Uterine atony was the most common indication for hysterectomy in primiparous, whereas placenta accreta was the most common in multiparas. Fifty-five (70%) of the women had a subtotal abdominal hysterectomy. Uterine atony was the major reason for total hysterectomies (19 out of 23, 83%). Nine of the 35 (26%) women with placenta previa without accreta underwent a total hysterectomy. Thirteen of 36 (36%) women with placenta previa with accreta underwent a total hysterectomy. No difference in the type of hysterectomy was noted when the indication was placenta previa with or without accreta (p=0.34). To prevent EPH, different pharmacologic agents and surgical procedures were used to stop bleeding including uterotonics in 60 (76%), oversewing bleeding points 8 (10%), use of uterine packs or/and balloon tamponade in 62 (78%), B Lynch suture in 4 (5%), uterine artery ligation in 50 (63%), and internal iliac artery ligation in 32 (41%), (Figure 2). The most common intraoperative complication was bladder injury in 18 (23%), followed by ureteric injury 2 (2%). Additional complications included vaginal cuff cellulitis/vault hematoma 6 (8%), disseminated intravascular coagulopathy 5 (6%), pulmonary edema in 2 (2%), deep vein thrombosis 1 (1%), and wound infection 1 (1%); Table 3. All women were actively managed with resuscitation and transfusions, the average numbers of blood and blood products transfused are presented in Table 4. The median number of packed red blood cell units transfused was 8 (range, 0-31). The average maternal length of postoperative stay in hospital was 16±14 days (range, 4-66 days) and the mean length of postoperative stay in the intensive care unit was 2.2±1.9 days (range 1-6 days). We had one case of maternal death (1%): para one woman with triplets who had PPH and hysterectomy and was complicated with disseminated intravascular coagulation. Twenty-two (28%) of delivered neonates were admitted to NICU, with one case of death (1%) (Table 3).

## Discussion

Although rare in modern obstetrics, EPH is a major surgery and is invariably performed in the presence of life-threatening hemorrhage during or immediately after abdominal or vaginal deliveries^(14)^. Modern obstetricians employ EPH when all conservative measures have failed to achieve hemostasis during life-threatening hemorrhage. The unplanned nature of the EPH surgery, the need for performing it expeditiously, and the acute loss of blood complicates the performance of EPH. Emergency PPH following a CS was first described by Porro and reported to be used to prevent maternal mortality due to postpartum hemorrhage^(6,7)^. Dubai, in the UAE, is a metropolitan city with more than 180 nationalities, lifestyle changes, and cultural differences which have transformed Dubai into a major healthcare hub. Our study included 168.293 deliveries performed in two major tertiary care hospitals in Dubai from 2000 to 2015. Both hospitals are tertiary, government-owned, and they serve as referral centers for many community-based hospitals and other nearby cities. Interestingly, despite the constant rise in CS rates (32% in the past four years) in DHA-governed hospitals, the EPH rate has remained low when compared with data from the region and global rates. Our study demonstrates an EPH incidence of 0.47 per 1000 deliveries (Table 1). The global reported impact of EPH varies from 0.24 to 8.9 per 1000 deliveries,^(8)^ ranging from 0.33 (Netherlands), 0.2 (Norway), 0.3 (Ireland), 0.63 (Saudi Arabia) and 1.2 to 2.7 per 1000 deliveries in the United States of America (USA)^(14)^. A difference in the incidence of EPH is noted following a vaginal delivery and CS, and is reported to be 0.1 to 0.3/1000 in vaginal births^(5,14)^. The incidence of EPH following CS varies widely between 0.17 and 8.7/1000 deliveries with a global rate of EPH ranging from 0.2 and 2.7 in 1000 deliveries^(11)^. This differs between countries with the lowest rates of 0.2 (Norway),^(5)^ 0.24 (China),^(15)^ 0.3 (Ireland),^(16)^ 0.36 (Turkey),^(17)^ and in countries with 1.9 (India),^(18)^ 1.39 (Iran),^(19)^ 1 (Kuwait),^(20)^ 0.5 [the Kingdom of Saudi Arabia (KSA)],^(21)^ 0.8 (Canada),^(14)^ and 1.2 to 2.7 (USA)^(22)^ per 1000 deliveries. CS deliveries have been on the rise in the past 20 decades throughout the world^(23)^. The reason for this significant rise is multifactorial: use of electronic fetal heart monitoring, risks and fear of litigation, payment schemes with cesarean deliveries being paid more, and increased convenience for both obstetricians and women^(24)^ are the main contributing factors. In fact, there is an increased demand of elective CS on maternal request^(25)^. Presently, we do not have clear data on how many of the CSs in UAE are based on a maternal request, but according to Hamilton et al.^(9)^ worldwide, the maternal request CS rate has been estimated between 12-15%. We have noted that although the total number of deliveries per year was decreasing in Dubai, the number of CSs has risen (Table 1). This increase of CS rate eventually causes an increase in abnormal placentation, and consequently an expected rise in the incidence of EPH^(11)^.It has been presented that women who had a history of placenta previa with a previous uterine scar had a 16% higher risk of hysterectomy compared with 3.6% in women with unscarred uteri^(5,14,21)^. In our series, 94% (74/79) of EPH cases were due to abnormal placentation, and there was a significant association between the number of previous CSs and abnormal placentation (p=0.007). Equal percentages were shared between placenta accreta and placenta previa without accreta as an indication for EPH (Table 2). This supports the fact that recognizing and assessing patients at risk and appropriate and timely intervention facilitates, and counseling women at risk enables better EPH outcomes. In this study, 70% of the EPHs were subtotal hysterectomies, and 30% were total, which is in line with a literature review^(8)^. The main reasons for preferring subtotal hysterectomy are less blood loss, reduction of operating time as well as postoperative complications. No evidence for a difference in the rates of incontinence, constipation, or sexual function between total and subtotal hysterectomy has been reported^(26)^. The most frequent indications of EPH in modern obstetrics are placenta accreta and uterine atony, respectively,^(8)^ and our observations in the UAE are consistent with this. 

Peripartum hysterectomy is associated with high complication rates, including a maternal mortality (range from 0 to 12.5% with a mean of 4.8%),^(14) ^and the death rate was close to 1% 1 (79) in our settings. Despite the high incidence of CSs and multiparity in UAE, the risk for EPH (0.47/1000) was lower in comparison with other countries in the region: 1.9 (India),^(18)^ 1.39 (Iran),^(19)^ 1 (Kuwait),^(20)^ and 0.5 (KSA)^(21)^. The results of the study are important in many ways and provide the first national estimate for EPH, especially post CS in the UAE. To the best of our knowledge, there have been no other studies conducted in the UAE to assess EPH rates in the context of CS. The data from this study can further enable the implementation of local guidelines to reduce CS, which might decrease the incidence of EPH. Patient participation and counseling can be encouraged to mitigate the risks of EPH in our local hospitals. We can expect to limit the number of CS through the reduction of unnecessary inductions of labor, encouragement of external cephalic version, allowing adequate time for labor initiation in the first stage, and reducing medicalized labor. We believe that patient-payer mechanisms should encourage normal delivery and that auditing indications for CS in public and private hospitals might also reduce the CS rate. It is anticipated that results from our study might be used by countries with a similar culture in the Eastern and Middle Eastern region of the world.

## Conclusion

The primary indication of the EPH was the abnormal placentation in a scarred uterus and uterine atony. The major method of prevention of EPH is to assess women’s risks and to reduce the number of CS deliveries, by limiting the rate of the primary cesareans. This is challenging in the UAE where the culture is associated with high gravidity and high parity. We recommend that the national agenda in the UAE should include public health actions that might assist in the reduction of primary and repeat CSs.

## Figures and Tables

**Table 1:**
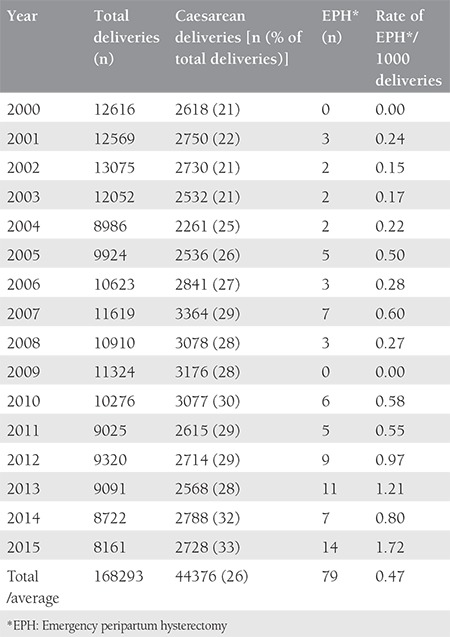
Rate of emergency peripartum hysterectomy by year in the dubai health authority

**Table 2:**
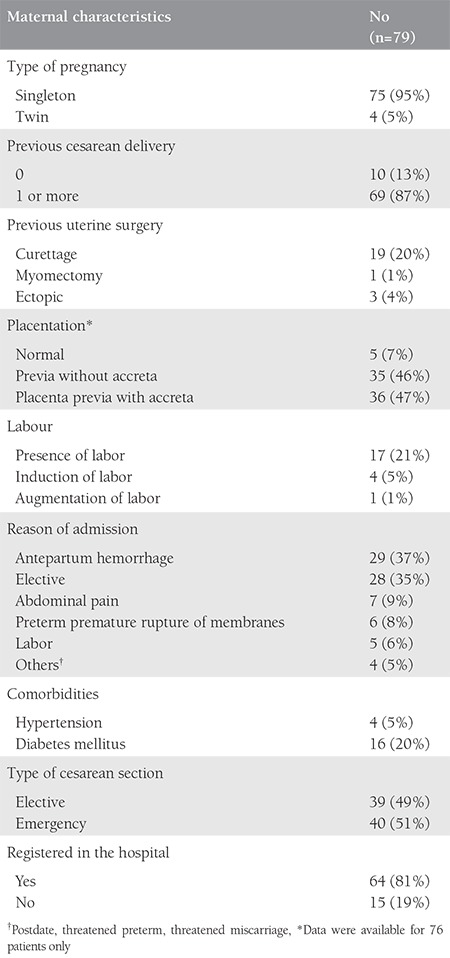
General characteristics of patients with peripartum hysterectomy

**Table 3:**
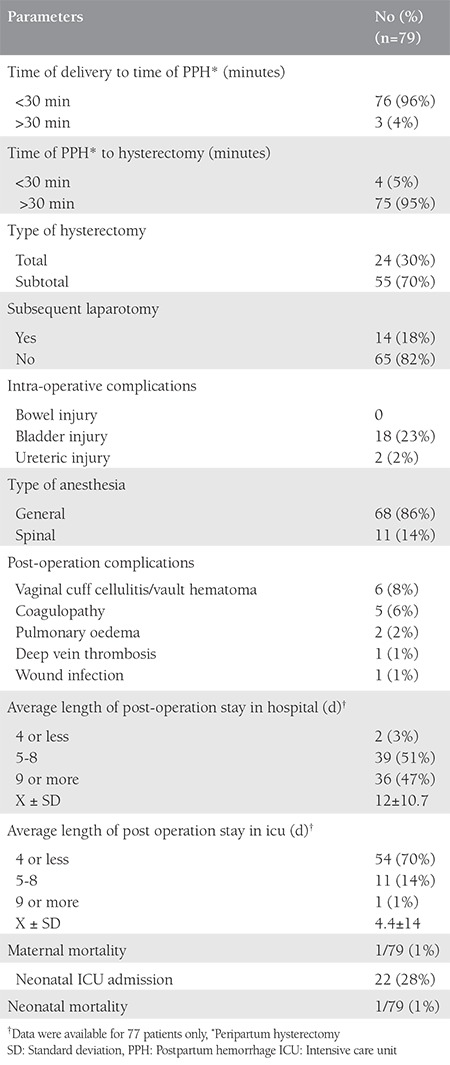
Distribution of mothers according to parameters related to their peripartum hysterectomy

**Table 4:**
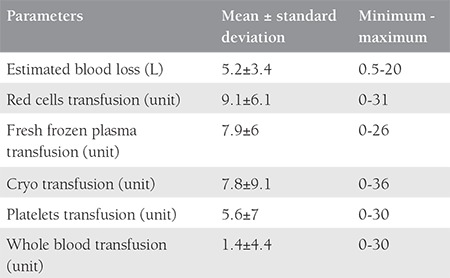
Quantity of blood and blood products transfused
